# Editorial: Exercise endocrinology: hormonal variations and relationships with acute and chronic exposure to exercise

**DOI:** 10.3389/fendo.2023.1344032

**Published:** 2023-12-20

**Authors:** Eugenia Murawska-Ciałowicz, Ana Filipa Silva, Luca Paolo Ardigò, Filipe Manuel Clemente

**Affiliations:** ^1^ Department of Physiology and Biochemistry, Wroclaw University of Health and Sport Sciences, Wroclaw, Poland; ^2^ Escola Superior Desporto e Lazer, Instituto Politécnico de Viana do Castelo, Rua Escola Industrial e Comercial de Nun’Álvares, Viana do Castelo, Portugal; ^3^ Sport Physical Activity and Health Research & Innovation Center (SPRINT), Viana do Castelo, Portugal; ^4^ Department of Teacher Education, NLA University College, Oslo, Norway

**Keywords:** physical exercise, clinical population, aerobic exercise, resistance-training, endocrinology

## Introduction

In this comprehensive exploration of physical exercise endocrinology, recent studies within the Research Topic, “*Exercise Endocrinology: Hormonal Variations and Relationships with Acute and Chronic Exposure to Exercise*,” converge to paint a nuanced picture of how different forms of exercise influence hormonal dynamics and, subsequently, impact various physiological processes. As we embark on this journey through the findings, it’s essential to recognize the interconnectedness of these studies, each shedding light on a different facet of the intricate relationship between exercise and hormones.

## Prediabetes and aerobic exercise

The systematic review and meta-analysis titled “*Effects of aerobic exercises in prediabetes patients*” (Wang et al.) lay the groundwork by investigating the influence of aerobic exercise on individuals with prediabetes. Examining outcomes such as BMI, FBG, 2hPG, and HbA1c in 815 prediabetic patients across 10 randomized controlled trials, the study establishes a foundation for understanding the positive impact of aerobic exercise on metabolic markers. This sets the stage for further exploration into how exercise, when sustained over time, can be a key player in managing prediabetes.

## Age matters: immunological response to moderate aerobic exercise

Moving forward, the study “*Exercise improves intestinal IgA production by T-dependent cell pathway in adult but not in aged mice*” (Hernandez-Urban et al.) delves into the immunological impact of moderate aerobic exercise, showcasing distinct responses in adult and aged mice. By uncovering how exercise influences IgA production through the T-dependent pathway, this study bridges the gap between age and exercise efficacy, highlighting the need for age-specific considerations in designing exercise interventions for immune system support.

## Exercise and fracture risk post-thyroidectomy

Transitioning into the realm of post-surgery care, the study “*Physical activity and reduced risk of fracture in thyroid cancer patients after thyroidectomy*” (Kim et al.) broadens the scope by investigating the fracture risk in individuals over 40 who underwent thyroidectomy for thyroid cancer. By analyzing a vast dataset of 74,774 subjects, the research unveils a significant reduction in fracture risk associated with regular exercise. This study not only emphasizes the importance of exercise in post-thyroidectomy care but also underlines its potential role in bone health across various contexts.

## Cognitive and biochemical benefits of nordic walking

Shifting gears, the study “*Nordic Walking training in BungyPump form improves cognitive functions and physical performance*” (Rodziewicz-Flis et al.) expands our understanding by exploring the holistic impact of a 12-week Nordic Walking training program on older adults. This intervention not only enhances cognitive functions and physical performance but also induces changes in biochemical profiles. The interconnectedness between physical activity, cognitive well-being, and biochemical responses suggests a multifaceted approach to exercise interventions for older populations.

## Deconstructing assumptions: serum and muscle steroids in resistance training

In a departure from conventional wisdom, the study “*Acute changes in serum and skeletal muscle steroids in resistance-trained men*” (Vechin et al.) challenges assumptions about the alignment of serum and muscle steroid concentrations in resistance-trained individuals. By investigating the acute hormonal response to resistance exercise, the findings prompt a reevaluation of our understanding of the dynamics between serum and muscle steroid concentrations, urging a more nuanced approach to studying the effects of resistance training.

## Exercise’s impact on skeletal health in lipodystrophy

Bringing our exploration to a close, the study “*Exercise Increases Bone in SEIPIN Deficient Lipodystrophy, Despite Low Marrow Adiposity*” (McGrath et al.) takes a unique stance by investigating the impact of exercise on skeletal health in the context of lipodystrophy. Despite the absence of white adipose tissue, exercise induces a significant increase in trabecular bone volume, suggesting the potential for exercise to play a crucial role in musculoskeletal health, even in conditions characterized by metabolic abnormalities.

As we navigate through these studies, the threads connecting them become apparent: exercise is not just a physical endeavor but a holistic intervention with far-reaching implications for health, touching on metabolism, immunity, bone health, cognition, and hormonal dynamics. This collective understanding paves the way for more targeted and effective exercise recommendations that consider the diverse physiological responses to different forms of physical activity.

## Author contributions

EM-C: Conceptualization, Writing – original draft, Writing – review & editing. AS: Conceptualization, Writing – original draft, Writing – review & editing. LA: Conceptualization, Writing – original draft, Writing – review & editing. FC: Conceptualization, Writing – original draft, Writing – review & editing.

